# Unraveling the Gene Regulatory Networks of the Global Regulators VeA and LaeA in Aspergillus nidulans

**DOI:** 10.1128/spectrum.00166-23

**Published:** 2023-03-15

**Authors:** Heungyun Moon, Mi-Kyung Lee, Ilhan Bok, Jin Woo Bok, Nancy P. Keller, Jae-Hyuk Yu

**Affiliations:** a Department of Plant Pathology, University of Wisconsin—Madison, Madison, Wisconsin, USA; b Department of Bacteriology, University of Wisconsin—Madison, Madison, Wisconsin, USA; c Biological Resource Center/Korean Collection for Type Cultures, Korea Research Institute of Bioscience and Biotechnology, Jeongeup, Republic of Korea; d Department of Biomedical Engineering, University of Wisconsin—Madison, Madison, Wisconsin, USA; e Department of Medical Microbiology and Immunology, University of Wisconsin-Madison, Madison, Wisconsin, USA; f Department of Systems Biotechnology, KonKuk University, Seoul, Republic of Korea; Universidade de Sao Paulo

**Keywords:** *Aspergillus*, VeA, LaeA, global regulator, development, metabolism, DNA binding motif, gene regulatory network

## Abstract

In the filamentous fungus Aspergillus nidulans, the *velvet* family protein VeA and the global regulator of secondary metabolism LaeA govern development and secondary metabolism mostly by acting as the VelB/VeA/LaeA heterotrimeric complex. While functions of these highly conserved controllers have been well studied, the genome-wide regulatory networks governing cellular and chemical development remain to be uncovered. Here, by integrating transcriptomic analyses, protein–DNA interactions, and the known A. nidulans gene/protein interaction data, we have unraveled the gene regulatory networks governed by VeA and LaeA. Within the networks, VeA and LaeA directly control the expression of numerous genes involved in asexual/sexual development and primary/secondary metabolism in A. nidulans. Totals of 3,190 and 1,834 potential direct target genes of VeA and LaeA were identified, respectively, including several important developmental and metabolic regulators such as *flbA*·*B*·*C*, *velB*·*C*, *areA*, *mpkB*, and *hogA*. Moreover, by analyzing over 8,800 ChIP-seq peaks, we have revealed the predicted common consensus sequences 5′-TGATTGGCTG-3′ and 5′-TCACGTGAC-3′ that VeA and LaeA might bind to interchangeably. These findings further expand the biochemical and genomic studies of the VelB/VeA/LaeA complex functionality in the gene regulation. In summary, this study unveils genes that are under the regulation of VeA and LaeA, proposes the VeA- and LaeA-mediated gene regulatory networks, and demonstrates their genome-wide developmental and metabolic regulations in A. nidulans.

**IMPORTANCE** Fungal development and metabolism are genetically programmed events involving specialized cellular differentiation, cellular communication, and temporal and spatial regulation of gene expression. In genus Aspergillus, the global regulators VeA and LaeA govern developmental and metabolic processes by affecting the expression of downstream genes, including multiple transcription factors and signaling elements. Due to their vital roles in overall biology, functions of VeA and LaeA have been extensively studied, but there still has been a lack of knowledge about their genome-wide regulatory networks. In this study, employing the model fungus A. nidulans, we have identified direct targets of VeA and LaeA and their gene regulatory networks by integrating transcriptome, protein–DNA interaction, and protein–protein interaction analyses. Our results demonstrate the genome-wide regulatory mechanisms of these global regulators, thereby advancing the knowledge of fungal biology and genetics.

## INTRODUCTION

Fungi are of great importance in human lives and environments by playing diverse roles in different aspects: the medical field (human pathogens and antibiotics producers), the food industry (fermentation and process), agriculture (pathogens and growth aids), and environmental recycling (decomposer). In nature, most filamentous fungi reproduce mainly through asexual sporulation, which generates multicellular asexual reproductive organs and nonmotile spores. In some fungi, this main reproductive system is tightly coupled with secondary metabolite production. Several studies in Aspergillus species have reported that developmental mutants, defective in sexual and/or asexual developments, coincidentally exhibited a loss of ability to produce mycotoxins such as sterigmatocystin (ST) and aflatoxin (AF) (1 to 3). This type of genetic link between development and metabolism has been observed in a variety of fungal species (1).

**FIG 1:**
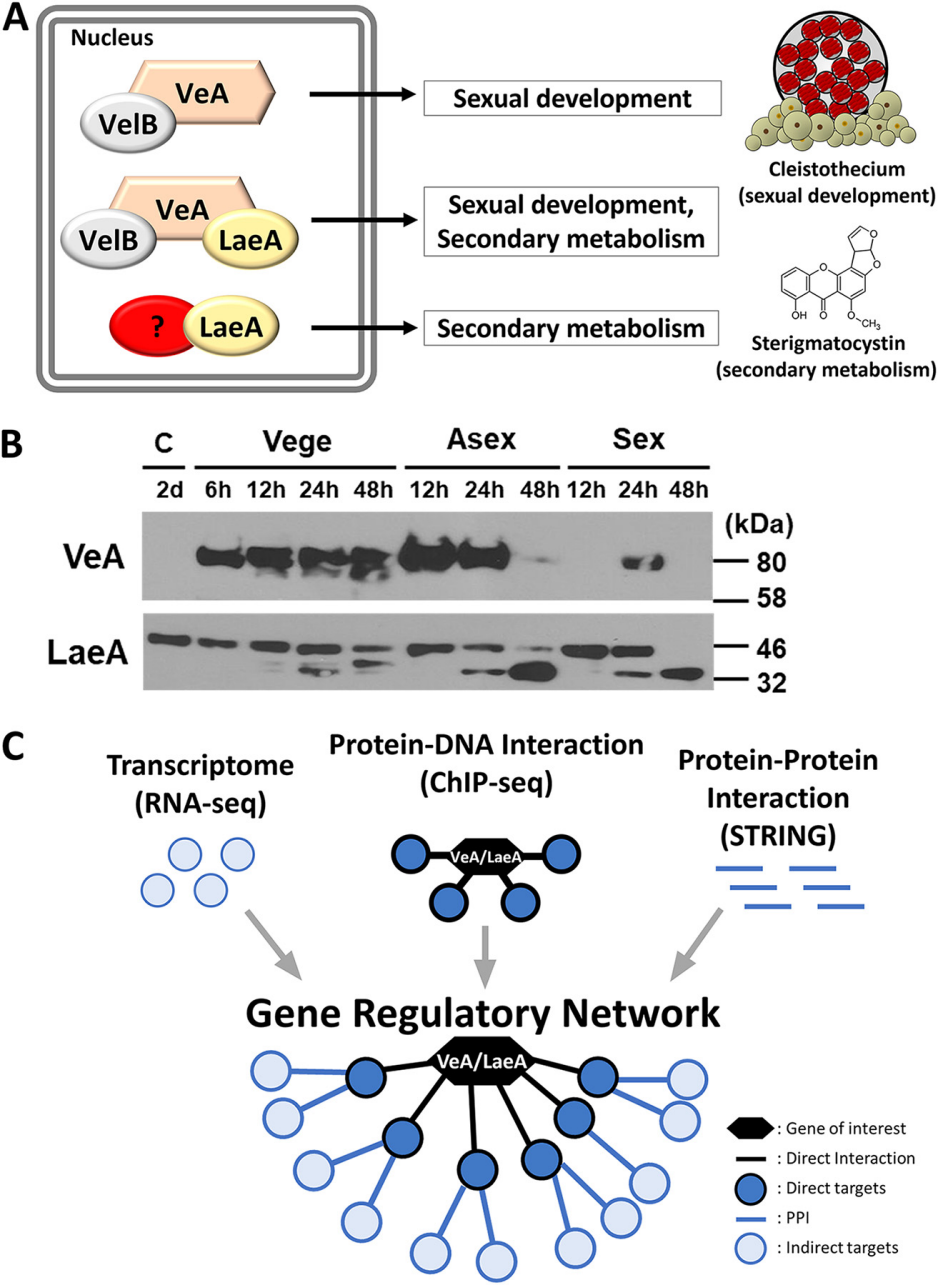
Overview of VeA- and LaeA-mediated regulation in A. nidulans and a schematic presentation of this study. (A) In the dark, VeA enters nucleus with the other *velvet* protein VelB and forms different *velvet* complexes, including the VelB/VeA/LaeA heterotrimer, which regulates sexual development and secondary metabolism. VeA is considered a major partner of LaeA, yet LaeA may also interact with other proteins for controlling the production of various secondary metabolites. (B) VeA and LaeA expression during the life cycle: conidia (C), vegetative growth (Vege), asexual (Asex), and sexual (Sex) development. (C) In this study, we have integrated transcriptomic profiling (RNA-seq), chromatin immunoprecipitation sequencing (ChIP-seq), and known protein–protein interaction (PPI; STRING) data to construct the gene regulatory networks.

In the ubiquitous fungal genus Aspergillus, a few global regulators govern development and metabolism at a bona fide upstream molecular level. These regulators directly and indirectly affect the expression of a vast array of genes, including transcription factors (TFs), playing different biological roles and processes. In the model filamentous fungus A. nidulans, the *velvet* family proteins (VeA, VosA, VelB, and VelC), LaeA, and NsdD are well-known global regulators of development and secondary metabolism (4 to 7), and due to their functions, VeA and LaeA have been extensively studied (Fig. 1A). Previous studies have revealed that VeA is a key light-dependent developmental regulator that activates sexual development yet represses asexual sporulation (8, 9). Once VeA enters the nucleus in the absence of light, it physically interacts with other regulators and forms diverse complexes, including the VelB/VeA/LaeA heterotrimer, governing fungal development and secondary metabolism (10, 11). Bok and Keller (5) identified and elucidated that LaeA is required for the production of several secondary metabolites (SMs) including ST, penicillin, and lovastatin, via conferring proper expression of corresponding SM biosynthetic gene clusters. Despite the pivotal regulatory roles of VeA and LaeA in Aspergillus biology, a genome-wide snapshot has not been established due to the complexity of the associated gene regulatory networks (GRNs).

In this study, we elucidate GRNs of VeA and LaeA, employing 24-h vegetatively grown cells of A. nidulans (Fig. 1B). Previous GRN studies have utilized multiple RNA-seq data sets to infer a GRN, but this type of GRN was unable to provide molecular evidence of TF and regulator interactions. On the other hand, our study overcomes this shortcoming by introducing physical protein–DNA and protein–protein interaction analyses. Genome-wide direct and indirect target genes of VeA and LaeA are determined by integrating RNA sequencing (RNA-seq) and chromatin immunoprecipitation followed by sequencing (ChIP-seq) analyses, and these target genes are subjected to the network analyses for constructing VeA- and LaeA-mediated GRNs by merging with reported protein–protein interaction data (STRING database) (Fig. 1C).

Within the VeA- and LaeA-mediated GRNs, we have revealed respective core sections demonstrating their central regulatory mechanisms in the genetic regulation of development and metabolism in A. nidulans. Furthermore, we propose a common gene regulatory network controlled by both VeA and LaeA and its core section, elucidating a potential universal GRN mediated by the VeA/LaeA complex.

## RESULTS

### Genome-wide transcriptomic profiling of *veA*-and *laeA*-null mutants in A. nidulans.

To understand the regulatory roles of VeA and LaeA in A. nidulans, we carried out genome-wide gene expression analyses of vegetatively grown cells (Vege, 24 h grown in the dark) of wild type (WT) and the Δ*veA* and Δ*laeA* (null) mutants. Totals of 29.74% (3,268/10,988) and 21.25% (2,338/10,988) of genes were differentially expressed in the Δ*veA* and Δ*laeA* mutant Vege, respectively. Among the differentially expressed genes (DEGs), 39% and 39.7% were upregulated, and 61% and 60.3% were downregulated in the Δ*veA* and Δ*laeA* Vege, respectively, suggesting that both VeA and LaeA tend to activate (rather than repress) the expression of diverse genes.

To gain an understanding of the functional roles of DEGs in Δ*veA* and Δ*laeA*, functional category enrichment analyses were performed utilizing gene ontology (GO) terms (Table S1 in the supplemental material). The results demonstrate that in the Δ*veA* Vege, upregulated genes are associated with primary housekeeping processes such as translation, peptide metabolic process, cellular amino acid metabolic process, cellular nitrogen compound biosynthetic process, and amide metabolic process, while downregulated genes were involved in secondary metabolic processes, including phenol-containing compound, organic heteropentacyclic compound, melanin, monodictyphenone, and toxin. Similar outcomes were observed in the Δ*laeA* Vege: upregulated genes were implicated in transmembrane transport, including xenobiotic, carbohydrate, and amide transports, some secondary metabolic processes, and nitrogen cycle metabolic process, and downregulated genes were mostly related to secondary metabolic processes, including austinol, ketone, and alkaloid. Taken together, these results suggest that both VeA and LaeA positively regulate secondary metabolism while repressing some primary metabolic and housekeeping processes in A. nidulans.

### Identification of potential direct targets of VeA and LaeA.

**FIG 2:**
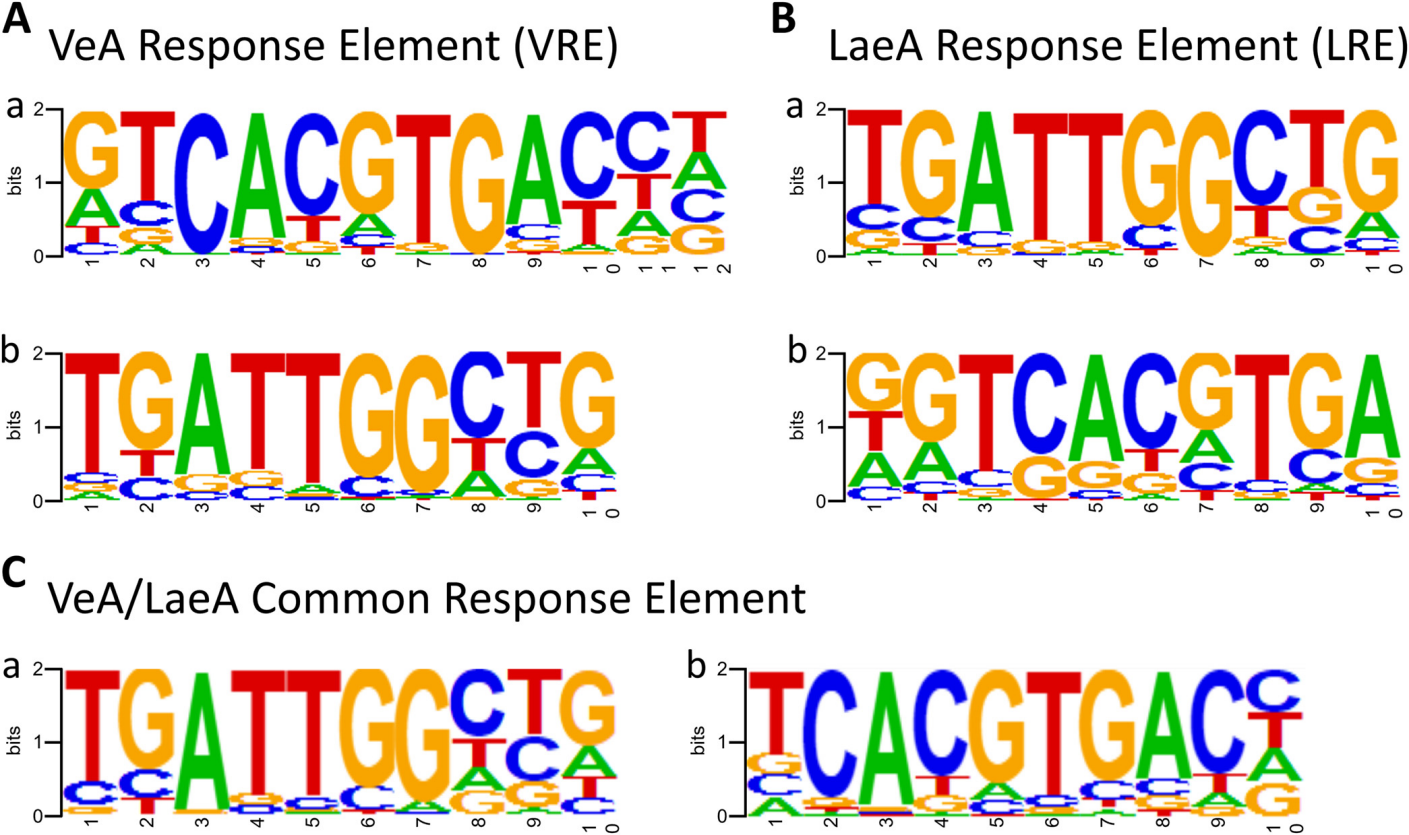
Identification of VeA and LaeA response elements. (A) Predicted VeA response elements (VREs) are 5′-GTCACGTGAC-3′ and 5′-TGATTGGCTG-3′. (B) Predicted LaeA response elements (LREs) are 5′-TGATTGGCTG-3′ and 5′-GTCACGTGA-3′. (C) The common (overlapped) response elements of VeA and LaeA are 5′-TGATTGGCTG-3′ and 5′-TCACGTGAC-3′. Through panels A to C, a is the first ranked response elements and b is the second ranked response elements from motif elicitation analysis.

To identify the putative direct target genes of VeA and LaeA, ChIP followed by high-throughput sequencing was carried out using anti-FLAG antibody and individual strains expressing the VeA or LaeA proteins with the FLAG epitope. Totals of 3,190 and 1,834 genes were identified as VeA and LaeA peak-associated genes, respectively, determined as putative direct targets in this study. Then, to identify consensus nucleotide sequences (response elements) of VeA and/or LaeA binding (VREs and LREs), totals of 5,502 and 3,333 peaks were subjected to the Homer *de novo* Motif Elicitation analysis, respectively (Fig. 2). Interestingly, we have found a high similarity between the predicted VREs and LREs. The top two predicted VREs were 5′-GTCACGTGAC-3′ (6.14% of input peaks with a *P* value of 1e-81) and 5′-TGATTGGCTG-3′ (16.52% of input peaks with a *P* value of 1e-65) (Fig. 2A), and the top two LREs were 5′-TGATTGGCTG-3′ (10.83% of input peaks with a *P* value of 1e-39) and 5′-GTCACGTGA-3′ (9.39% of input peaks with a *P* value of 1e-35) (Fig. 2B). Both 5′-TGATTGGCTG-3′ and 5′-TCACGTGA-3′ sequences appeared in predicted VeA and LaeA binding motifs. As VeA and LaeA are known to function as a complex, we speculated that these response elements are potential binding motifs of the VeA/LaeA complex, we thus decided to perform an additional *de novo* Motif Elicitation analysis on the overlapping peaks between the VeA and LaeA ChIP-seq data. We found a total of 1,186 overlapping peaks, and they were subjected to the Motif Elicitation analysis. The top two predicted response elements of overlapping peaks were 5′-TGATTGGCTG-3′ (26.31% of input peaks with a *P* value of 1e-47) and 5′-TCACGTGAC-3′ (14.92% of input peaks with a *P* value of 1e-40) (Fig. 2C), suggesting that both 5′-TGATTGGCTG-3′ and 5′-TCACGTGAC-3′ are potential binding motifs of VeA and LaeA, and they may recognize these sequences in the complex form.

**FIG 3:**
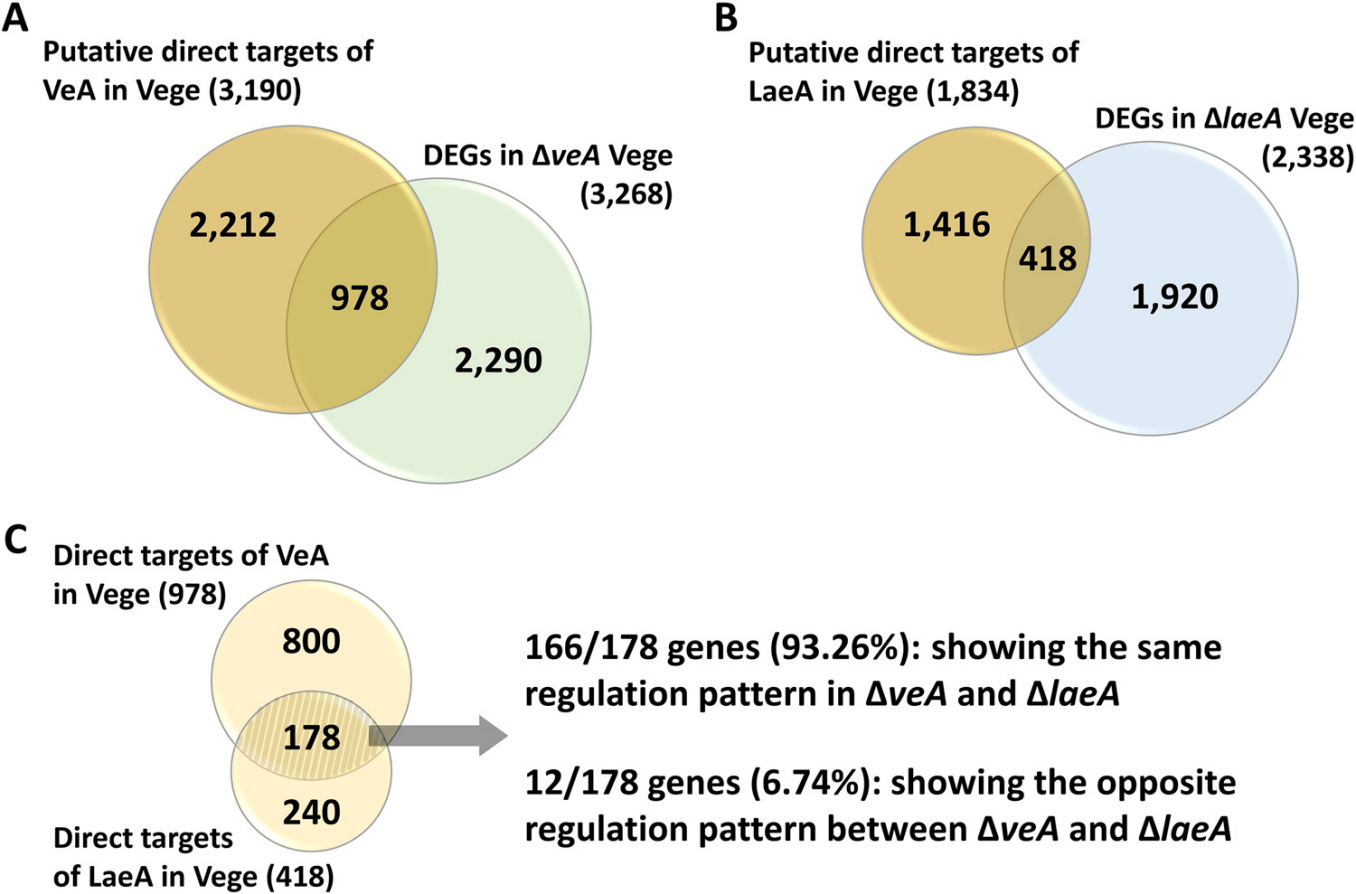
Identification of VeA and LaeA direct targets in A. nidulans. (A) The Venn diagram showing the number of putative direct targets of VeA and DEGs in the Δ*veA* Vege. The overlapped part in the Venn diagram indicates the number of direct targets of VeA. (B) The Venn diagram showing the number of putative direct targets of LaeA and DEGs in the Δ*laeA* Vege. The overlapped part in the Venn diagram indicates the number of direct targets of LaeA. (C) The Venn diagram depicting the number of overlapped direct targets of VeA and LaeA. The 166 genes might be under the direct regulatory control of the VeA/LaeA complex.

We then compared the results of the ChIP-seq and RNA-seq analyses to identify genes that might be under the direct regulatory control (direct targets) of VeA and LaeA. Direct target genes are determined when their promoter regions are bound by VeA or LaeA, and their gene expression levels are altered in Δ*veA* or Δ*laeA*. Totals of 978 (8.9% of 10,988 genes; Fig. 3A) and 418 (3.8% of 10,988 genes; Fig. 3B) genes were identified as VeA and LaeA direct targets, respectively. Additionally, 178 common direct targets of VeA and LaeA were identified (Fig. 3C). Interestingly, 93.26% (166/178) of these common targets displayed the same regulation pattern in Δ*veA* or Δ*laeA*; 63 genes were upregulated, and 103 genes were downregulated in both null mutants (Fig. 3C). Collectively, these results indicate that VeA and LaeA directly regulate a large array of target genes involved in development and metabolism and the 166 common genes may be under the direct regulatory control of the VeA/LaeA complex.

To further investigate the roles of direct targets of VeA, LaeA, or both in Aspergillus biology, functional category analysis was carried out by determining GO terms (Table S2). Among the VeA direct target genes, several genes involved in general translation and metabolism of peptide, cellular nitrogen compound, and amide were upregulated, whereas the expression of genes related to glycogen metabolism, cellular glucan metabolic process, syncytium formation, and cleistothecium formation were downregulated in Δ*veA*. These results suggest that VeA might directly activate polysaccharide metabolic processes and sexual development, yet negatively regulate primary metabolic processes, including translation, peptide, amide, and nitrogen. Some LaeA direct target genes associated with cellular response to diverse stressors, carbohydrate transmembrane transport, glycolysis, and NADH regeneration displayed higher expression levels in Δ*laeA* compared to WT. On the other hand, the mRNA levels of LaeA direct target genes involved in syncytium formation, anatomical structure formation, and diverse regulations such as cell differentiation, asexual sporulation, and sexual development were decreased in Δ*laeA*. These results indicate that LaeA directly activates diverse developmental processes, while directly repressing stress responses, carbohydrate transmembrane transports, and some processes in glycolysis. The upregulated VeA/LaeA common target genes in both the Δ*veA* and Δ*laeA* Vege are associated with cellular response to stressors, regulation of defense response, and generation of precursor metabolites and energy. On the other hand, the downregulated VeA/LaeA common targets in both the Δ*veA* and Δ*laeA* Vege are associated with regulating syncytium formation, cell-to-cell fusion, cell differentiation, glycogen biosynthesis, manganese ion homeostasis, and RNA interference, implying that by acting in concert, VeA and LaeA might directly activate fungal growth and cellular development, whereas they inhibit some defense mechanisms, including responses to stressors in general.

### VeA-mediated GRN.

To elucidate the detailed genome-wide regulatory roles of VeA and LaeA, the network analyses were conducted by integrating the results of ChIP-seq and RNA-seq and the protein–protein interaction database (STRING).

**FIG 4:**
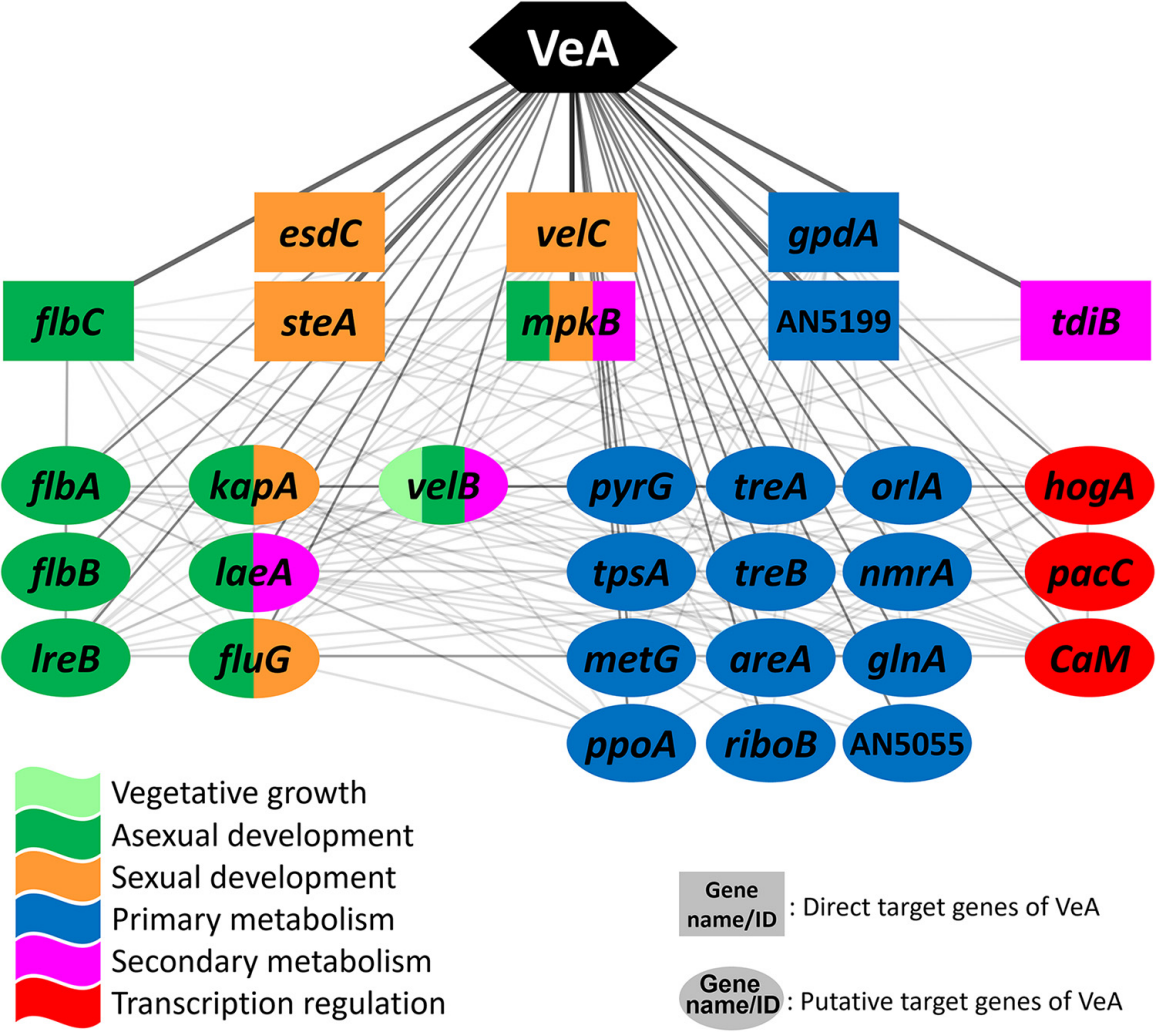
The core section of VeA-mediated GRN. In the network, genes in rectangles are direct targets, and those in ovals are putative direct targets of VeA. The colors represent predicted functions as shown. Each line indicates an interaction between two genes/proteins.

From the ChIP-seq and RNA-seq analyses, the 978 direct target genes and 2,212 putative direct targets of VeA were selected and overlaid with the *A. nidulans* protein–protein interaction (PPI) database, which led to the VeA-mediated GRN consisting of 2,210 genes (nodes) and 83,844 interactions between genes (edges). This GRN enabled us to uncover the interactions between direct and putative direct target genes of VeA. To elucidate the central regulatory function of the VeA-mediated GRN, we analyzed the first neighbors of VeA within the network and have found that the core section is composed of 8 direct targets and 22 putative direct targets, including several well-known genes encoding developmental and metabolic regulators of Aspergillus such as *flbA*·*B*·*C*, *velB*·*C*, *mpkB*, *laeA*, *areA*, and *hogA* (Fig. 4).

### LaeA-mediated GRN.

**FIG 5:**
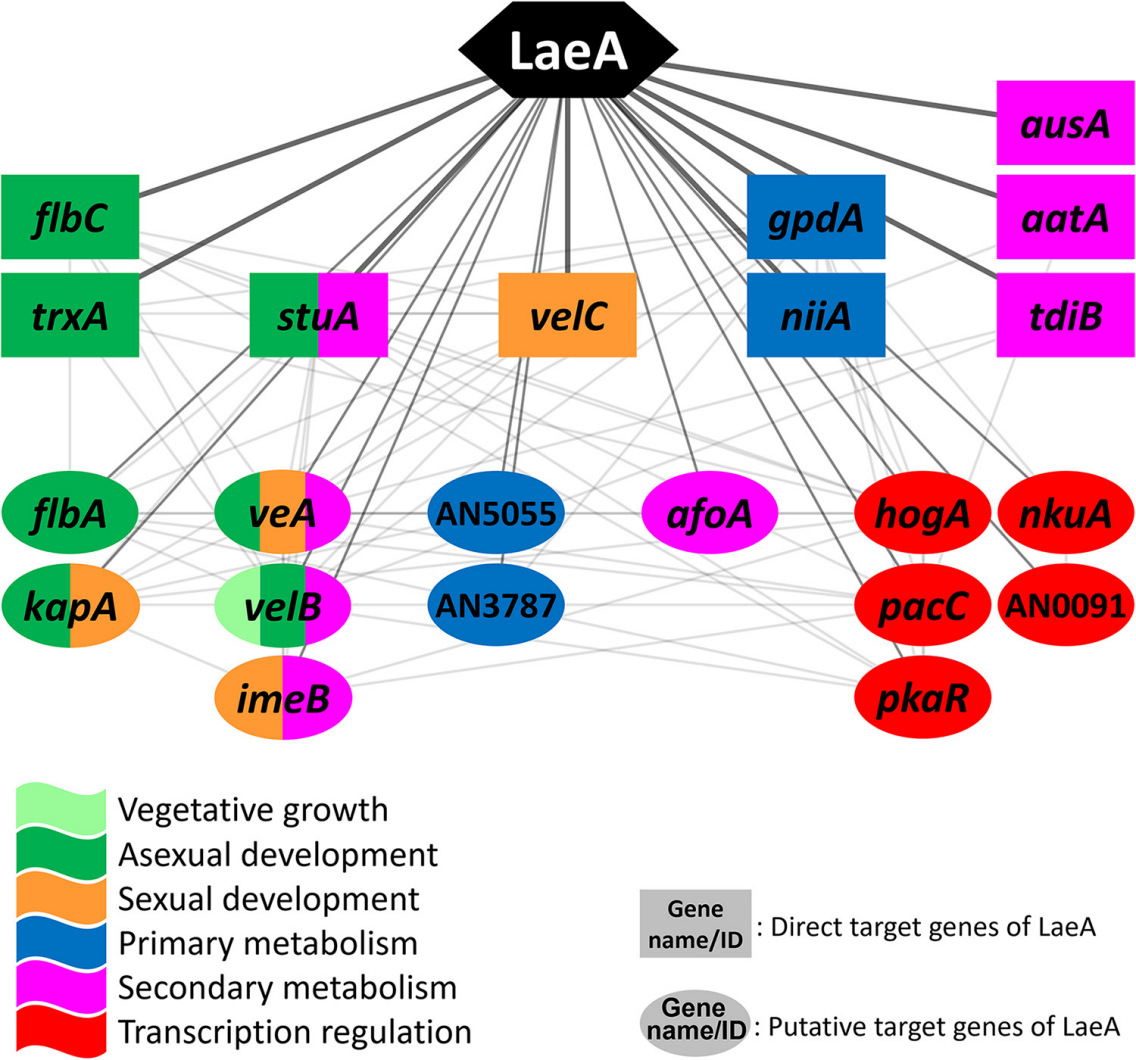
The core section of LaeA-mediated GRN. In the network, genes in rectangles are likely direct targets, and those in ovals are putative direct targets of LaeA. The colors represent predicted functions as shown. Each line indicates an interaction between two genes/proteins.

To perform the network analysis on LaeA, the 418 direct target genes and 1,416 putative targets of LaeA were selected from the RNA-seq and ChIP-seq analyses. We then combined the target genes of LaeA with the A. nidulans PPI database (STRING), resulting in the formation of LaeA-mediated GRN consisting of 1,206 nodes and 24,334 edges. We have elucidated the core section of LaeA-mediated GRN by applying the same method utilized in the VeA network analysis, which consists of 9 direct targets and 13 putative direct targets, including *flbA*, *flbC*, *kapA*, *trxA*, *veA*, *velB*, *velC*, *stuA*, *niiA*, *hogA*, and *pkaR* (Fig. 5). In the core section of the LaeA GRN, the 10 genes, *flbA*, *flbC*, *kapA*, *velB*, *velC*, AN5055, *gpdA*, *tdiB*, *hogA*, and *pacC*, also appear in the core section of the VeA GRN, suggesting that the VeA/LaeA complex may coregulate these key genes of development and metabolism in A. nidulans.

### Common GRN controlled by both VeA and LaeA.

**FIG 6:**
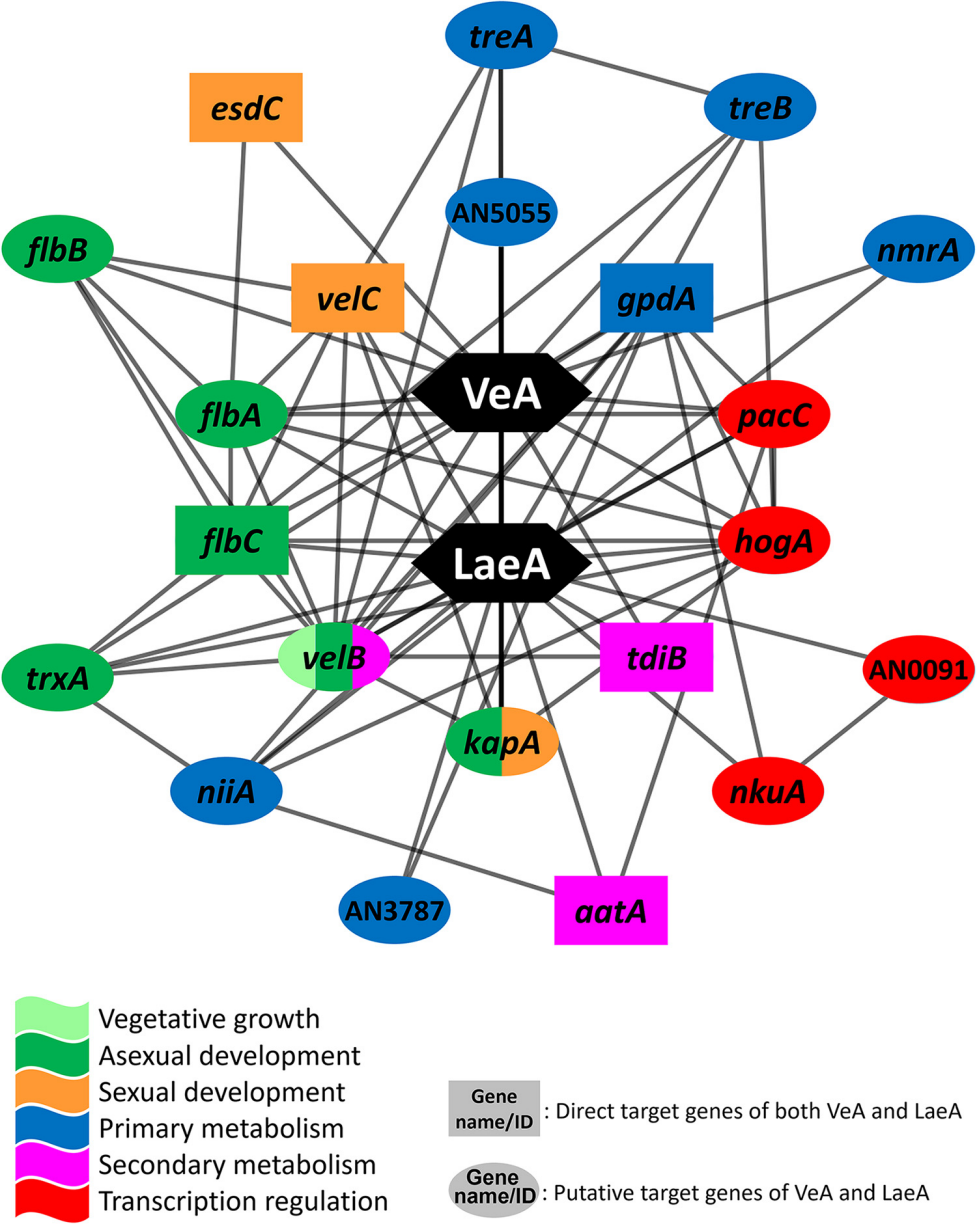
The core section of common GRN controlled by both VeA and LaeA. Genes in rectangles are likely direct targets of both VeA and LaeA, and genes in ovals are either likely or putative direct targets of VeA and LaeA.

To further understand a putative regulatory mechanism of the VeA/LaeA complex, we aimed to identify the common GRN of both VeA and LaeA in A. nidulans. While the common target genes of VeA and LaeA might vary from the actual direct targets of the VeA/LaeA complex, we presumed that the common target list shares high similarities with the actual targets of VeA/LaeA based on the RNA-seq, ChIP-seq, and motif analyses. To construct the common GRN, the ChIP-seq data of VeA and LaeA and the A. nidulans PPI database were integrated; a total of 1,084 putative common direct targets were identified by overlapping 3,190 and 1,834 putative direct target genes of VeA and LaeA, respectively. These 1,084 putative common targets then were overlaid with the A. nidulans PPI database, resulting in the formation of the common GRN, which is likely controlled by both VeA and LaeA, consisting of 693 nodes and 9,467 edges. To elucidate the core section of the common GRN, the same method was applied, and 21 genes, including 6 likely direct targets and 15 putative direct targets, were identified (Fig. 6). As we combined two different networks here, the definitions of “direct target” and “putative direct target” slightly differ from previous ones; only direct targets of both VeA and LaeA are indicated as direct targets, otherwise determined as putative direct targets in the common GRN.

In the core section of the common GRN, the *flbA*, *flbC*, *velB*, *velC*, *kapA*, AN5055, *gpdA*, *tdiB*, *pacC*, and *hogA* genes are commonly found in both core sections of VeA and LaeA GRNs. Interestingly, *esdC* and *aatA*, direct targets of both VeA and LaeA, appeared in the core section of the common network, but they appeared only in the core sections of VeA and LaeA GRNs, respectively, suggesting asymmetrical roles of individual proteins in the VeA/LaeA complex. In summary, these results imply that a majority of VeA and LaeA regulatory pathways is likely to be governed by the VeA/LaeA complex in A. nidulans.

## DISCUSSION

The global regulators VeA and LaeA govern developmental and metabolic processes in many (if not all) Aspergillus species. While regulatory roles of VeA and LaeA have been extensively studied, there has been a lack of knowledge about their genome-wide regulatory networks. In this study, we have identified the likely and putative direct targets of VeA and LaeA and their GRNs by integrating RNA-seq, ChIP-seq, and PPI analyses in A. nidulans (Fig. 1C). Previous GRN studies have relied on overlaying multiple RNA-seq results (single omics layer) to infer GRNs of interest, but this method has a critical limitation, that it cannot provide physical evidence of interaction and regulation for various TFs and regulators. Thus, further studies such as quantitative PCR and electrophoretic mobility shift assay (EMSA) are required to verify these GRNs (12). However, to make up for the imperfection of the single omics layer approach, we herein introduce network-based multilayered analyses, including physical protein DNA (ChIP-seq) and reported protein–protein interaction analyses in this study. Due to recent advances in diverse powerful omics tools, studies combining multiple omics layers became available and have shown that they yielded clearer snapshots of complex systems (13, 14). It is hoped that this study facilitates our understanding of Aspergillus GRN complexity.

The *veA* and *laeA* genes and their respective proteins are constitutively expressed during vegetative growth with distinct cellular localization patterns: LaeA is constitutively nuclear, while VeA is mostly localized in the nucleus in the dark and partially nuclear under the light (11, 15). Thus, we expected that in our 24-h Vege samples grown in the dark, most of the LaeA and VeA proteins are in the nucleus forming the VelB/VeA/LaeA heterotrimer complex and actively controlling target genes. Although we concluded that Vege 24 h is the most appropriate condition to investigate the targets of VeA and LaeA based on their biology and protein expression patterns throughout the life cycle (Fig. 1B), target genes of VeA and LaeA may vary to some extent depending on cell types (asexual and sexual cells) and growth conditions (time points and the presence of light). To understand the complete list of VeA and LaeA target genes, further studies in other conditions will be necessary.

Our functional-enrichment analyses suggested not only consistent results with known regulatory roles but also unexplored characteristics of VeA and LaeA. Enriched terms of DEGs demonstrate the broad effects of VeA and LaeA in Aspergillus biology; most downregulated genes in the Δ*veA* and Δ*laeA* Vege are associated with SM biosynthesis, whereas most upregulated genes in Δ*veA* and Δ*laeA* are related to primary metabolism (Table S1). In our GO term analyses on direct targets of these proteins, similar to the results from those of DEGs, the upregulated direct target genes in the Δ*veA* and Δ*laeA* Vege are associated with mostly primary metabolic processes. However, the downregulated direct targets in Δ*veA* are involved in growth, sexual development, and metabolic processes of complex polysaccharides, whereas the downregulated direct targets in Δ*laeA* are associated with the regulation of asexual and sexual developmental processes (Table S2). These results indicate that VeA and LaeA may play a crucial role in primary metabolism. Previous studies mostly focused on their functions in development and secondary metabolism, with little understanding of the underlying effects of VeA and LaeA in primary metabolism. Our findings suggest the necessity of investigating the roles of VeA and LaeA in controlling primary metabolic changes coupled with cellular and secondary metabolic development in A. nidulans. In addition, while LaeA is considered a global regulator of secondary metabolism, its effects on development need to be further investigated. In A. nidulans, the Δ*laeA* mutants show a similar radial growth on solid medium but exhibit distinct developmental patterns compared to WT: greatly reduced conidia production in the light but enhanced cleistothecia formation in the dark (15). Moreover, in other Aspergillus species, the roles of LaeA in development are also well described (16, 17). In conjunction with our GO term analyses, these together suggest that LaeA is a key upstream regulator of Aspergillus development. Besides, the VeA/LaeA common direct targets upregulated in both Δ*veA* and Δ*laeA* are mostly associated with fungal stress responses. Baidya et al. (18) reported that VeA plays a vital role in the regulation of oxidative stress response in Aspergillus flavus. Collectively, these results suggest that A. nidulans VeA and LaeA may play a key role in properly controlling fungal stress response. It is noteworthy that mutational inactivation of VeA leads to elevated production of pigment (19).

LaeA encodes a nuclear protein that contains an S-adenosylmethionine (SAM) binding motif found in histone methyltransferases and arginine methyltransferases, yet it lacks other conserved components of methyltransferases such as the SET (suppressor of variegation, enhancer of zeste, trithorax) domain and the double-E loop (5). As conventional methyltransferases, including LaeA, do not have a known DNA-binding domain, it is plausible that their interaction with chromatin would require other protein(s) that can interact with DNA and methyltransferases. From the ChIP-seq and motif elicitation analyses in this study, we have revealed that the majority of LREs precisely match with VREs (Fig. 2) and that many direct targets are shared between LaeA and VeA. These results demonstrate that a major partner protein of LaeA is VeA, which in turn enables LaeA to confer site-specific gene regulation. However, we hypothesize that there are additional partners of LaeA, as 40.9% (750/1,834 genes) of LaeA putative direct targets are not in the list of the VeA targets.

The renowned gene network database STRING (https://string-db.org/) provides known PPI information of all proteins in A. nidulans. The definition of PPI was limited to physical interaction in the early 2000s; however, it has been expanded as defined through seven evidence channels: experiments, database, text mining, gene coexpression, neighborhood, fusion, and co-occurrence. Based on these channels, the STRING database has generated a combined score for an interaction, which can be referred to all types of interactions such as protein–protein, protein–DNA, and gene–gene (20). The GRNs proposed in this study are defined as a protein–protein interaction network (PPIN). Utilizing a PPIN is suitable to perform a genome-wide network analysis. However, it does not provide a directionality of regulation between genes (or proteins), or a type of protein such as TF, unlike a traditional GRN. From the STRING database, we obtained 67 and 75 reported PPIs that VeA and LaeA have with other genes or proteins in A. nidulans, respectively, which constitute less than 1.2% of the VeA and LaeA interactions that we are proposing in this study (Fig. S1). These indicate that VeA- and LaeA-mediated regulatory mechanisms have been underexplored and that our study provides novel interaction information of VeA and LaeA. Through the network analyses, we have found that VeA and LaeA directly regulate multiple upstream regulators that govern various biological functions: development, metabolism, signaling, and transcription regulation, including MAPK and PKA.

In the core section of VeA-mediated GRN (Fig. 4), the *fluG*, *flbA*, *flbB*, and *flbC* are required for the proper transition from hyphal growth to conidiophore development (21). They act as upstream activators of *brlA*, which encodes C_2_H_2_ zinc finger TF initiating asexual development (conidiation) (22, 23). Moreover, the *lreB* (light response) gene, encoding a putative zinc-finger TF, is involved in the morphological and physiological differentiation of A. nidulans. The LreB protein is known to interact with VeA, FphA, and LreA and form a large protein complex in the nucleus, which plays a crucial role in red- and blue-light responses (24). Thus, our findings suggest that VeA represses conidiation by likely controlling the upstream developmental activators and interacting with blue- and red-light sensors. Furthermore, some VeA direct targets such as *velC*, *steA*, and *esdC* are necessary for proper sexual development. VeA is known to positively regulate sexual development in that the deletion of *veA* resulted in the absence of cleistothecia formation, even under sexual development-promoting conditions, while the overexpression of *veA* led to opposite phenotypes (25). The deletion of *velC*, one of the *velvet* family genes, resulted in reduced production of sexual fruiting bodies (cleistothecia), whereas the overexpression of *velC* led to enhanced formation of cleistothecia (4). The *steA* null mutant exhibited the complete absence of cleistothecium production (26). The *esdC* (early sexual development) gene is necessary for proper sexual fruiting body formation; however, its overexpression did not enhance this process. Throughout developmental stages, VeA is known to positively regulate the expression of *esdC* (27). In this study, we found that both VeA and LaeA bind to the promoter regions of the *esdC* gene, and its expression level is significantly downregulated in both Δ*veA* and Δ*laeA*. Along with the roles of these genes, the expressions of *velC*, *steA*, and *esdC* were all downregulated in the Δ*veA* Vege, implying that VeA activates sexual development by positively regulating key genes responsible for the sexual structure formation in A. nidulans. Moreover, the *kapA*, *velB*, and *laeA* genes appeared in the core section of the VeA GRN. KapA is the importin-α, which mediates the nuclear import pathway of the VeA/VelB heterodimer (10). Once the VeA/VelB complex enters the nucleus, VeA interacts with the nuclear protein LaeA, forming the VelB/VeA/LaeA heterotrimeric complex in the dark; this complex plays crucial roles in sexual development and secondary metabolism (11). Similarly, MpkB, a mitogen-activated protein kinase, affects diverse biological processes: germination and conidiation through regulating the expression of *vosA* and *brlA* as well as secondary metabolism through controlling the expression of *laeA* and secondary metabolism gene clusters, including *aflR* and *tdiB* (28, 29). Interestingly, a large portion of the core network was composed of primary metabolism-related genes: gluconeogenesis/glycolysis (*gpdA*), trehalose metabolism (*tpsA*, *treA*, *treB*, *orlA*), amino acid metabolism (*metG*, *glnA*), and nitrogen metabolism (*areA*, *nmrA*). Among these genes, *areA* encodes a GATA-type TF, which activates expression of genes necessary for nitrogen acquisition. These results suggest that VeA affects a broad spectrum of primary metabolic processes. The *hogA* gene encodes a mitogen-activated protein kinase, which plays a vital role in the high-osmolarity glycerol (HOG) response MAPK signaling pathway. In response to fludioxonil and osmotic stress, HogA activates the expression of the *atfA* gene encoding a bZip-type TF, which in turn alters the expression of numerous downstream genes involved in osmotic stress and fludioxonil responses (30). Taken together, the network analysis of the VeA-mediated GRN demonstrates that VeA governs Aspergillus biology by directly regulating specific key genes of conidiation, sexual development, primary metabolism, and secondary metabolism.

In the core section of LaeA-mediated GRN (Fig. 5), the *trxA* gene encodes thioredoxin A containing a thioredoxin active site motif (WCGPC). By working together with the thioredoxin reductase (TrxR), thioredoxin A plays a significant role in redox regulation. These thioredoxin systems not only coordinate protein disulfide reduction, sulfur assimilation, detoxification of reactive oxygen species, and redox regulation of enzymes but also affect growth and development of A. nidulans (31). The StuA (stunted locus A) is a TF classified as a spatial modifier of conidiophore morphogenesis. The deletion of *stuA* resulted in the production of greatly shortened conidiophores, with a lack of normal metulae and phialides (32). The effect of StuA in secondary metabolism of A. nidulans has not been characterized yet, but in other Aspergillus species, including A. fumigatus, the StuA orthologs regulate the expression of secondary metabolite biosynthetic genes, implying a possible role of StuA in the regulation of A. nidulans secondary metabolism (reviewed in reference 33). The *imeB* gene encodes a protein kinase necessary for light-mediated inhibition of sexual development and mycotoxin production. The *imeB* null mutant exhibited reduced growth but greatly enhanced production of fertile cleistothecia under the light condition, where the sexual development usually does not take place. Moreover, ImeB is required for the proper expression of the ST biosynthetic gene cluster (34). In addition to the genes involved in Aspergillus development, several genes associated with primary and secondary metabolism are found in the LaeA core network: *gpdA* (gluconeogenesis/glycolysis), *niiA* (nitrogen utilization), *afoA* (asperfuranone), *ausA* (austinol/dehydroaustinol), *aatA* (penicillin), and *tdiB* (asterriquinone). Furthermore, some genes that function at the upstream level of diverse biological processes, such as *hogA*, *pkaR*, *nkuA*, and AN0091 (an ortholog of Saccharomyces cerevisiae DOT1), are found. HogA and PkaR are critical components of HOG MAPK and the PKA pathway, respectively. NkuA is the homolog of the human KU70 essential for DNA nonhomologous end-joining during double-strand break repair. As the deletion of *nkuA* drastically reduces the frequency of nonhomologous integration of designated DNA fragments during fungal transformation, it is widely used for gene-targeting techniques (35). The function of AN0091 has not been identified in A. nidulans yet, but Liang et al. (36) revealed that the DOT1 ortholog in A. flavus has a H3K79-specific histone methyltransferase activity that plays a vital role in heterochromatin formation, which in turn affects development, AF production, and virulence. Taken together, these results demonstrate that LaeA directly controls the expression of upstream genes in development, metabolism, and general transcription regulation in A. nidulans.

These GRN analyses may provide possible explanations at the genetic level for the previous cellular and chemical phenotypic alterations that occurred by the deletion or overexpression of *veA* and *laeA* genes in A. nidulans. For example, Δ*veA* mutants displayed repressed transcription of the ST-specific regulatory gene *aflR* and significantly decreased ST production regardless of the presence of light (11, 37). In this study, we found that the expression of 8 genes, including *aflR*, that make up the ST biosynthetic gene cluster, was significantly downregulated in Δ*veA*, being consistent with the previous findings. In addition, the core sections shed light on the regulatory roles of VeA and LaeA in primary metabolism, which have not been thoroughly investigated yet. Although most genes associated with primary metabolism in the core sections are well characterized, AN5055 and AN5199 are not functionally characterized yet even though they seem to play an important role in both VeA- and LaeA-mediated GRNs. Both AN5055 and AN5199 are predicted to encode methionine aminopeptidases showing 66% and 69% amino acid (aa) similarity, respectively, to human methionine aminopeptidase 1 (MetAP1). While they share 73% similarity with S. cerevisiae MAP1, they are different from MetAP2 and MAP2. Methionine aminopeptidases cleave the initiator methionine residue from newly synthesized proteins and thus are essential components of the N-terminal methionine excision pathway. This posttranslational modification is known as an essential process conserved from eubacteria to higher eukaryotes. In yeast, the deletion of both *MAP1* and *MAP2* is lethal, while the *Δmap1* single mutant exhibits greatly reduced growth, and the *Δmap2* mutant displays no distinct phenotypes (reviewed in reference 38). By using the protein–protein Basic Local Alignment Search Tool (BLAST) at the NCBI website (http://www.ncbi.nlm.nih.gov/blast), we have found that orthologs of AN5055 and AN5199 are present in 71 and 78 Aspergillus species, with over 59.84% and 67.52% aa identity, respectively, suggesting a conserved and potentially important role of these proteins in aspergilli. Given the foreseen crucial role and prevalence of AN5055 and AN5199 in Aspergillus species and other fungi such as S. cerevisiae (data not shown), understanding their actual functions in Aspergillus development and metabolism will improve our knowledge of fungal biology.

In conclusion, this study unravels the VeA- and LaeA-mediated GRNs and elucidates genome-wide regulatory mechanisms of these global regulators, thereby advancing the knowledge of fungal biology and genetics.

## MATERIALS AND METHODS

### Aspergillus strains and culture conditions.

Aspergillus strains used in this study are listed in Table S3. The fungal strains were inoculated into liquid or solid 1% glucose minimal medium (GMM) with appropriate supplements and incubated at 37°C. To obtain the conidia sample, approximately 10^5^ spores were spread onto solid GMM and incubated at 37°C for 2 days. The conidia were collected in phosphate-buffered saline (PBS) and counted using a hemocytometer. To collect vegetatively growing cells (Vege), 5 × 10^5^ conidia/mL were inoculated into 100 mL liquid GMM and incubated at 37°C, 220 rpm in the dark. To obtain asexually and sexually developing cells, induction of asexual development or sexual development was carried out as previously described (39). Samples were collected at designated time points.

### Construction of *veA*::FLAG and *laeA*::FLAG strains.

Sexual crosses of A. nidulans strains were conducted as described in Pontecorvo et al. (40). *laeA*::FLAG (TJW143) and *veA*::FLAG (TJW191) strains were generated using TNO2A7 and RJMP1.31 by inserting 3× FLAG-Afribo at the C terminus *laeA* and *veA* ORF (open reading frame) using modified double-joint (DJ) PCR, respectively (41). The *laeA*::FLAG and *veA*::FLAG transformants were confirmed by PCR and Southern blot (data not shown). Then, TJW143.2 (*laeA*::FLAG) and TJW191.2 (*veA*::FLAG) strains were sexually crossed with RDIT2.1 and RTMH207.13 to obtain RJW302.11 and RJW324.3 strains, respectively. The recombinants were confirmed by Southern blotting (Fig. S2) and Western blot (Fig. S3). Primers used in this study are listed in Table S4.

### Nucleic acid isolation and manipulation.

The oligonucleotides used in this study are listed in Table S4. Genomic DNA isolation was carried out as described in Lee et al. (42). A loopful of conidia (103 to 104/loop) from a solid culture were inoculated into 10 mL of liquid GMM on a sterile plate and incubated at 37°C for 12 to 15 h. Then, semitransparent mycelial mat was collected, squeeze-dried, and freeze-dried. Freeze-dried fungal tissues were ground by using a motor-spatula tool until they turned into a fine powder, and high-quality genomic DNA was isolated. Genomic DNA and total RNA isolation were carried out as previously described (39, 43).

### Protein extraction and Western blot analysis.

Western blot analyses of VeA and LaeA were performed as described in Jeong and Yu (44). Briefly, 2-day-old conidia (2 × 10^8^), vegetatively grown cells (6, 12, 24, 48 h), asexually developing cells (12, 24, 48 h), and sexually developing cells (12, 24, 48 h) were collected and resuspended in the lysis buffer containing a 1× protease inhibitor cocktail. Then, samples were homogenized by a mini-beadbeater with 0.5 mm zirconia/silica beads. Protein concentrations were measured by the Bio-Rad Protein Assay (Bio-Rad). Approximately 15 μg of total proteins per lane were separated on 4 to 12% gradient SDS-PAGE (Bio-Rad) gel and transferred onto immobilon-P PVDF membrane (Millipore). The membrane was blocked with a blocking buffer, incubated with the monoclonal anti-FLAG antibody produced in mice (clone M2, Sigma-Aldrich), and then incubated with HRP-conjugated anti-mouse IgG (Millipore). The membrane was developed using Amersham enhanced chemiluminescence detection reagents (GE Healthcare).

### RNA sequencing analysis.

Total RNA samples were submitted to the Novogene company (Beijing, China) for sample quality check, library preparation, and mRNA sequencing. The quality of total RNA was validated thoroughly in multiple experimental confirmations using 1% agarose gel electrophoresis, Qubit 3.0 fluorometer (Thermo Fisher), and Agilent 2100 Bioanalyzer. During this step, RNA concentration (≥20 ng/μL), purity (OD260/280 > 2.0), and integrating number (RIN ≥ 6.3) were verified to proceed to the library preparation. A strand-specific library was prepared using an Illumina TruSeq strand-specific RNA sample preparation system. The DNA library of 250 to 300 bp insert size was constructed and sequenced using an Illumina NovaSeq 6000 platform with a 150-bp paired-end sequencing strategy. Over 3.3 × 10^7^ high-quality reads with 5 × 10^9^ clean bases and less than 0.03% base error rate for all samples were achieved. The genome and gene annotations were downloaded from NCBI (https://www.ncbi.nlm.nih.gov/search/all/?term=GCF_000149205.2+).

Mapping of the clean reads to the genome was carried out using Hisat2 version 2.1 (45). Over 84.9% of total reads were mapped to the genome. Gene expression level was processed using FeatureCounts version 1.5.0 (46) and quantified as FPKM values covering all genes in each sample. For the differential expression analysis, DESeq2 version 1.6.3 (47) was used to determine significantly differentially expressed genes. Briefly, genes were considered DEGs when they exhibited an adjusted *P* value of < 0.05 and more than 2-fold changes of increase or decrease. The default parameter settings were used for programs unless indicated specifically.

### Chromatin immunoprecipitation sequencing analysis.

To collect samples for ChIP-seq analysis, vegetatively grown cells (24 h) of RDIT9.32, RJW324.3, and RJW302.11 were cross-linked with 1% formaldehyde, resuspended in lysis buffer, and homogenized by a mini-beadbeater with 0.5 mm zirconia/silica beads. The lysates were then sonicated for five to seven cycles (60 s on, 60 s off) with a sonicator to achieve 150- to 200-bp size DNA fragments (44). After centrifugation, the lysates were diluted in the ChIP dilution buffer and applied for ChIP assays using the MAGnify Chromatin Immunoprecipitation System (Invitrogen) following the manufacturer’s instruction with a modest modification. The diluted chromatin extracts were incubated with 1 μg of mouse monoclonal anti-FLAG antibody (Sigma-Aldrich). As negative controls, the chromatin extracts were reacted with 1 μg of anti-rabbit IgG. Initial input DNAs before immunoprecipitation were used as positive controls. The enriched DNA fragments were retrieved and used as a template for ChIP-seq.

ChIP DNA samples were sent to ProteinCT (Madison, WI) for library preparation and sequencing. DNA libraries were prepared using the TruSeq ChIP Library Preparation kit (Illumina) and sequenced using an Illumina HiSeq2500 platform. More than 8 million reads per sample were achieved. The read sequences were mapped to the genome using bowties2 (48), and Homer (version 4.11) (49) was utilized to call peaks. To make peak calls, high sensitivity settings were used: more than 2-fold changes and a *P* value less than 0.001. Identification of VeA and LaeA direct targets was done by selecting genes, in which peaks are found in their promoter regions within the 1.5-kb upstream range from the translation start site (TSS). The response elements of VeA and LaeA were determined using the Homer *de novo* motif enrichment. To figure out the overlapping peaks between VeA and LaeA ChIP-seq data, the “merge peaks” function of Homer was used. Overlapping peaks were determined when the centers of VeA- and LaeA-associated peaks were located within 100 bp. Then, the overlapping peaks were analyzed using the Homer *de novo* motif enrichment to predict the VeA/LaeA common response elements.

### Functional enrichment analysis.

Gene ontology enriched terms were identified using the tools available at FungiDB (50). The parameters used in this study were biological process for the ontology, no limit to GO Slim terms, and a 0.05 *P* value for the cutoff. Then enriched terms were sorted by *P* values in ascending order.

### Gene regulatory network analysis.

We defined VeA- and LaeA-mediated GRNs as a protein–protein interaction network (PPIN), which consists of VeA or LaeA, its direct targets identified by overlapping ChIP-seq and RNA-seq analyses, and its putative direct target genes identified from ChIP-seq exclusively. The known PPI information of A. nidulans was obtained from the gene network database STRING (version 11.5) (51), by matching the protein ID and gene ID using the “protein.aliases” table provided by the database. We selected out edges provided by the PPI database with a threshold for confidence score of 150 and having both nodes belonging to the direct and/or putative direct target genes. These nodes and edges were used to construct a GRN. To investigate the core sections of the GRNs, we analyzed GRNs by applying the “guilt-by-association” principle, which was fulfilled by examining the first neighbors of the gene/protein of interest in the network. For the visualization of the core sections, direct targets were presented in a rectangle shape, and putative direct targets were presented in an oval shape. Then shapes were colored based on their functional categories: vegetative growth (pale green), asexual development (green), sexual development (deep saffron), primary metabolism (blue), secondary metabolism (magenta), and transcription regulation (red). The network visualization was performed using Cytoscape software (version 3.9.1) (52).

### Data availability.

All RNA-seq and ChIP-seq data that are relevant to this study are freely available from the NCBI Gene Expression Omnibus database (GSE217820) (veA and laeA RNA-seq, accession number GSE217815; VeA and LaeA ChIP-seq, accession number GSE217819). The GRN files are available in https://drive.google.com/drive/folders/16LGmluyCBXjyfApKRMVxCQE9_hPYgxWv?usp=sharing.
