# Supplementary material for: Unraveling the Gene Regulatory Networks of the Global Regulators VeA and LaeA in Aspergillus nidulans
Source: Microbiol Spectr. 2023 Mar 15;11(2):e00166-23. doi: 10.1128/spectrum.00166-23 (PMC10101098; doi:10.1128/spectrum.00166-23)
Supplement: Supplemental file 1 — Supplemental material. Download spectrum.00166-23-s0001.docx, DOCX file, 1.5 MB [file spectrum.00166-23-s0001.docx]

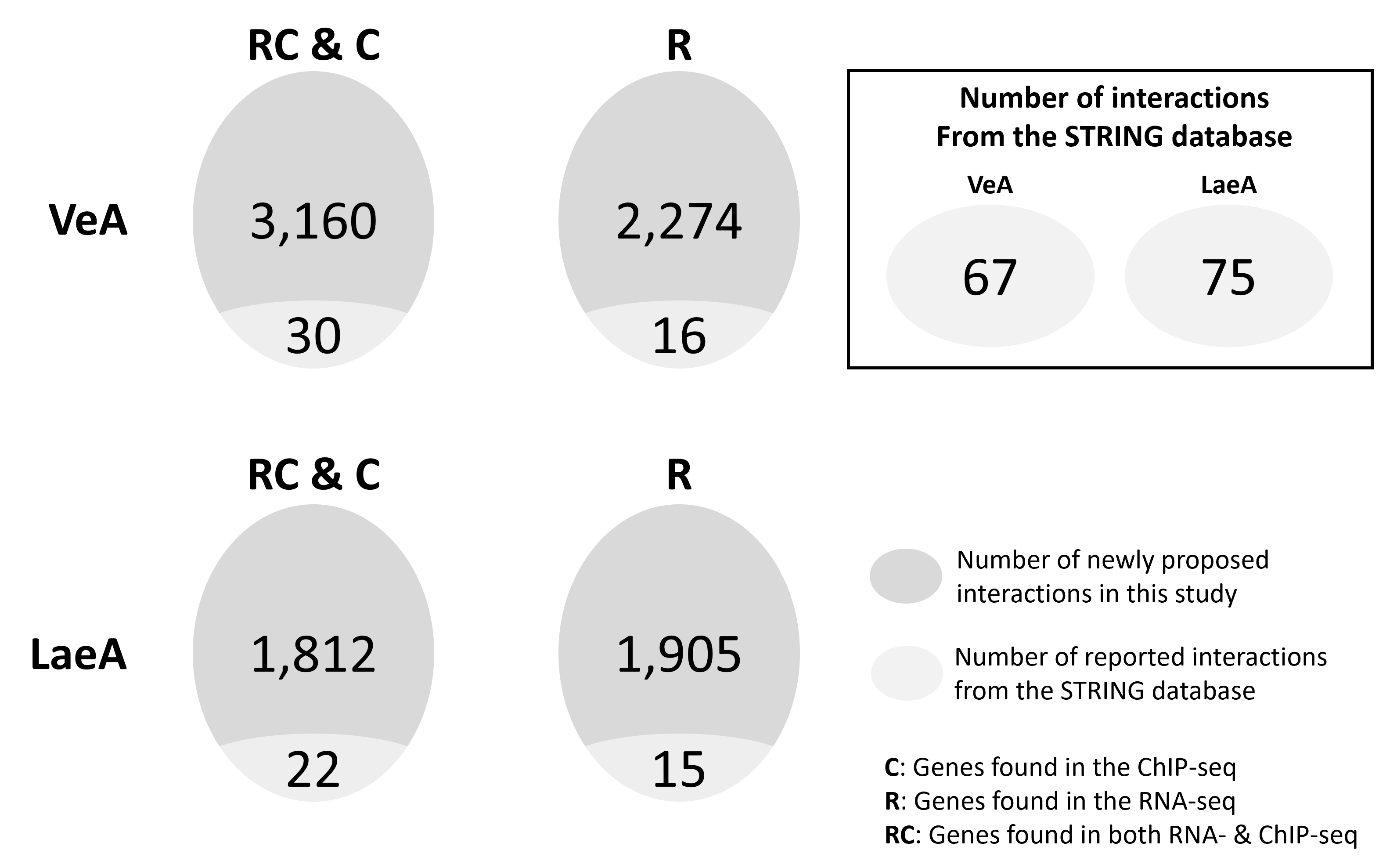
**Figure S1.** The known interactions of VeA and LaeA from the STRING database and novel interactions of VeA and LaeA proposed in this study.

**
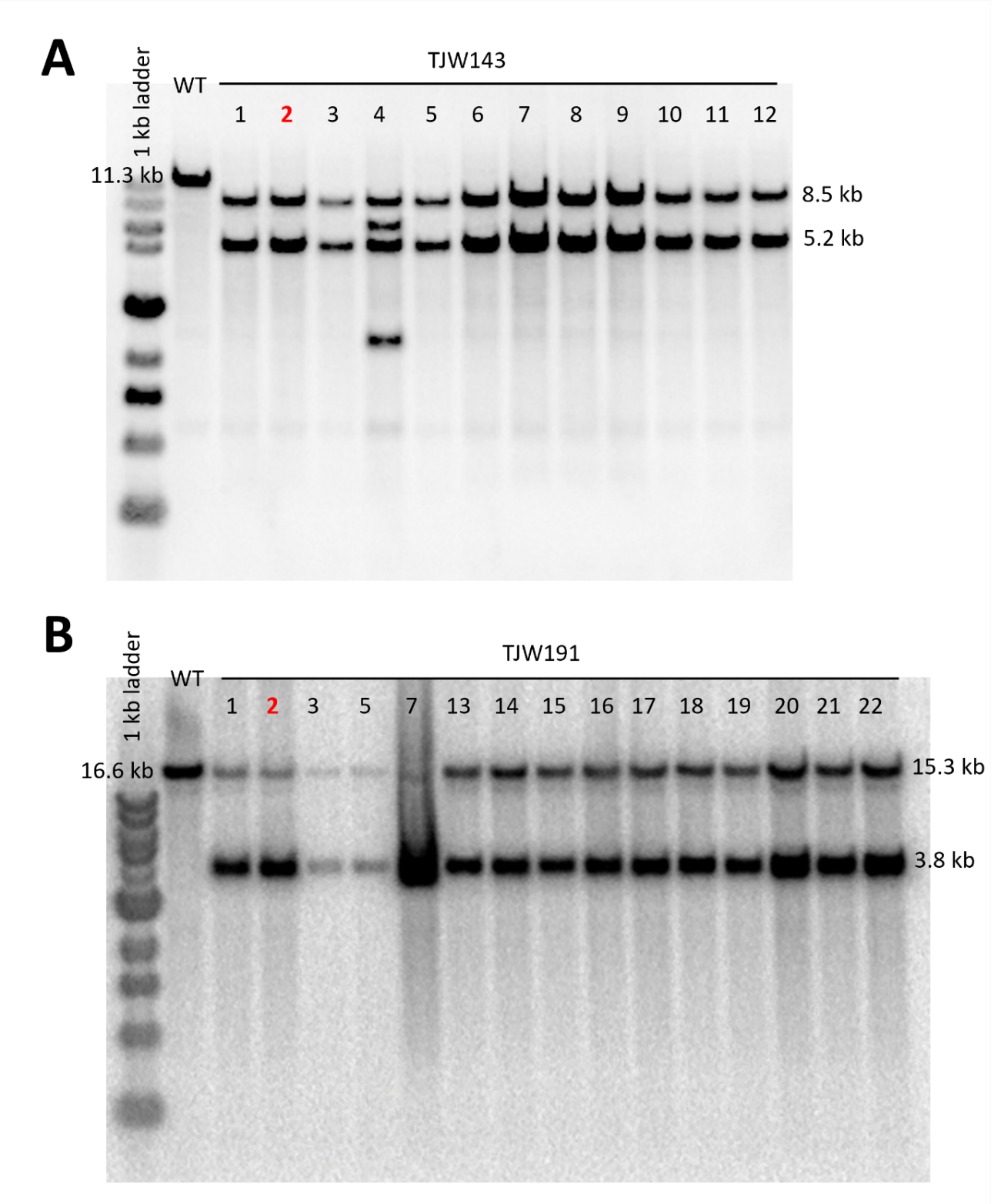
**

**Figure S2.** Southern blot confirmation of *laeA*::FLAG (A) and *veA*::FLAG (B) strains. (A) Genomic DNA was digested by *Eco*RI. Wild type (WT, 11.3 kb), and *laeA*::FLAG (5.2 and 8.5 kb). TJW143.2 was chosen for the subsequent experiment. (B) Genomic DNA was digested by *Xba*I. Wild type (WT, 16.6 kb), and *veA*::FLAG (3.8 and 15.3 kb). TJW191.2 was chosen for the subsequent experiment.

**
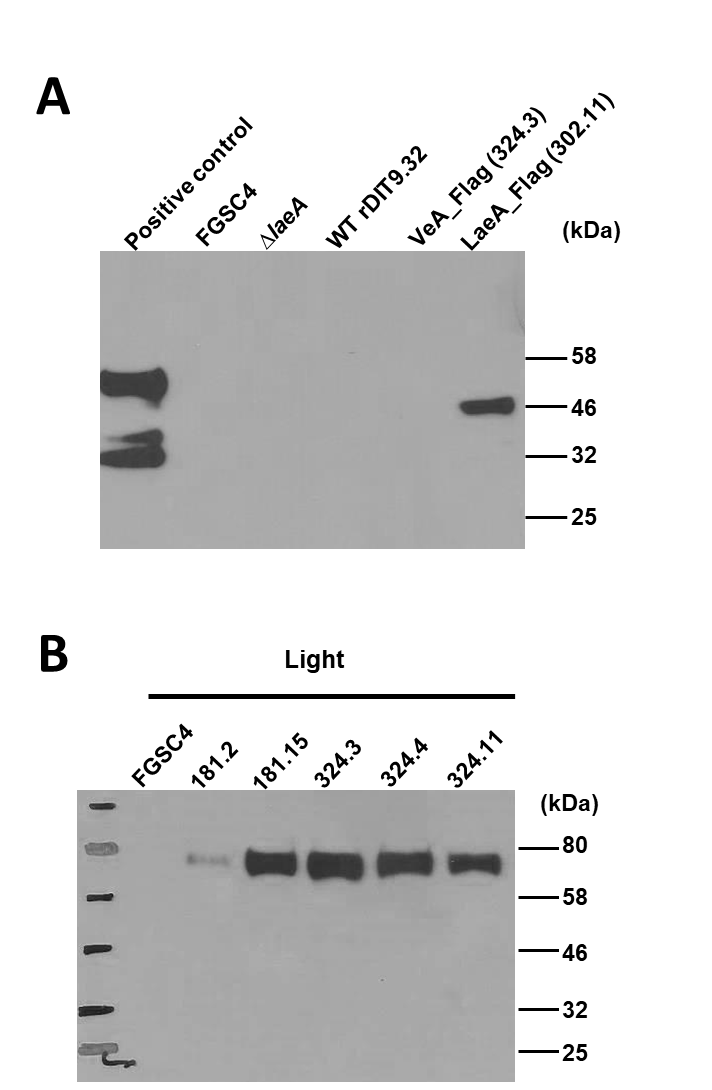
**

**Figure S3.** Western blot confirmation of *laeA*::FLAG and *veA*::FLAG strains. (A) Western blot analysis for LaeA using anti-FLAG antibody in *C’laeA* (RJW302.11) strains. A strain expressing FLAG tag-fused proteins was used as a positive control. Strains were cultured on solid GMM at 30 °C for 2 days and conidia samples were subjected to the blotting. The predicted molecular weight of LaeA::3xFLAG is 45.96 kDa (B) Western blot analysis for VeA using anti-FLAG antibody in C’*veA* (RJW324.3) strains. Strains were cultured on liquid GMM at 30 °C for 2 days. The predicted molecular weight of VeA::3xFLAG is 62.32 kDa.

| **Table S1.** Functional enrichment analyses on DEGs in ∆*veA* and ∆*laeA* Vege   \| **DEGs in ∆*veA* Vege** \| **DEGs in ∆*laeA* Vege** \| \| --- \| --- \| \| ***Biological processes associated with up-regulated genes in ∆veA (1,274) or ∆laeA (928)*** \| \| \| translation \| transmembrane transport \| \| peptide metabolic process \| secondary metabolic process \| \| amide metabolic process \| xenobiotic transmembrane transport \| \| organonitrogen compound metabolic process \| amide transport \| \| cellular nitrogen compound biosynthetic process \| reactive nitrogen species metabolic process \| \| organic substance biosynthetic process \| methionine biosynthetic process \| \| alpha-amino acid biosynthetic process \| nitrogen cycle metabolic process \| \| cellular amino acid metabolic process \| carbohydrate transmembrane transport \| \| ***Biological processes associated with down-regulated genes in ∆veA (1,994) or ∆laeA (1,410)*** \| \| \| secondary metabolic process \| secondary metabolic process \| \| phenol-containing compound metabolic process \| phenol-containing compound metabolic process \| \| organic heteropentacyclic compound metabolic process \| monodictyphenone metabolic process \| \| benzene-containing compound metabolic process \| benzene-containing compound metabolic process \| \| monodictyphenone metabolic process \| alkaloid metabolic process \| \| melanin metabolic process \| acetate metabolic process \| \| toxin metabolic process \| ketone metabolic process \| \| aflatoxin metabolic process \| austinol metabolic process \| |
| --- | --- | --- | --- | --- | --- | --- | --- | --- | --- | --- | --- | --- | --- | --- | --- | --- | --- | --- | --- | --- | --- | --- | --- | --- | --- | --- | --- | --- | --- | --- | --- | --- | --- | --- | --- | --- | --- | --- |

| **Table S2.** Functional enrichment analyses on VeA and/or LaeA direct target genes   \| **VeA direct targets** \| **LaeA direct targets** \| **VeA/LaeA common targets** \| \| --- \| --- \| --- \| \| ***Biological processes up-regulated in ∆veA (417), ∆laeA (176), or both Vege (63)*** \| \| \| \| translation \| cellular response to stressors \| cellular response to stressors \| \| organonitrogen compound metabolic process \| carbohydrate transmembrane transport \| purine-containing compound metabolic process \| \| peptide metabolic process \| methionine biosynthetic process \| regulation of defense response \| \| amide metabolic process \| NADH regeneration \| modulation by symbiont of host defense response \| \| cellular nitrogen compound metabolic process \| canonical glycolysis \| adhesion of symbiont to host \| \| cellular biosynthetic process \| adhesion of symbiont to host \| generation of precursor metabolites and energy \| \| ***Biological processes down-regulated in ∆veA (561), ∆laeA (242), or both Vege (103)*** \| \| \| \| glycogen metabolic process \| regulation of cell differentiation \| syncytium formation \| \| glucan metabolic process \| syncytium formation \| cell-cell fusion \| \| syncytium formation \| regulation of sexual sporulation \| regulation of cell differentiation \| \| cleistothecium formation \| regulation of asexual sporulation \| glycogen metabolic process \| \| polysaccharide biosynthetic process \| anatomical structure formation involved in morphogenesis \| manganese ion homeostasis \| \| transmembrane transport \| cell-cell fusion \| production of siRNA involved in RNA interference \| |
| --- | --- | --- | --- | --- | --- | --- | --- | --- | --- | --- | --- | --- | --- | --- | --- | --- | --- | --- | --- | --- | --- | --- | --- | --- | --- | --- | --- | --- | --- | --- | --- | --- | --- | --- | --- | --- | --- | --- | --- | --- | --- | --- | --- | --- | --- |

| **Table S3.** *Aspergillus* strains used in this study   \| **Strain name** \| **Relevant genotype** \| **Reference** \| \| --- \| --- \| --- \| \| RDIT9.32 \| *A. nidulans* wild type \| [1] \| \| TNO2A7 \| *pyroA4, riboB2, nku70, veA1* \| [2] \| \| RDIT2.1 \| *metG1* \| [2] \| \| RTMH207.13 \| *pyrG89, veA1* \| [2] \| \| TJW143.2 \| *laeA::*FLAG*::AfriboB, pyroA4, nku70, veA1* \| this study \| \| TJW191.2 \| *veA::*FLAG*::AfriboB, pyroA4, nku70* \| this study \| \| RJW112.2 \| *∆veA::argB* \| [3] \| \| RJW41.A \| *methG1; ∆laeA::methG; veA* \| [3] \| \| RJW324.3 \| *veA::*FLAG*::AfriboB* \| this study \| \| RJW302.11 \| *laeA::*FLAG*::AfriboB, veA* \| this study \| |
| --- | --- | --- | --- | --- | --- | --- | --- | --- | --- | --- | --- | --- | --- | --- | --- | --- | --- | --- | --- | --- | --- | --- | --- | --- | --- | --- | --- | --- | --- | --- | --- | --- | --- |

| **Table S4.** Oligonucleotides used in this study   \| Primer Name \| Sequence (5’ → 3’) \| Purpose \| \| --- \| --- \| --- \| \| laeAFlag5F \| AGTCCATCACTGAACGAGAGCC \| LaeA::FLAG tag \| \| laeAFlag5R \| CCAGCGCCTGCACCAGCTCCGGCACCTCTTAATGGTTTCCTAGCCTGGTATATGTGC \| LaeA::FLAG tag \| \| FlagF \| GCCGGAGCTGGTGCAGGCGCTGGAGCCGACTACAAAGACCATGACGGTGATTATAAAG \| LaeA::FLAG tag \| \| FlagjointR \| AAGGGCGAATTCCAGCACACTGG \| LaeA::FLAG tag \| \| FlagriboF \| CCAGTGTGCTGGAATTCGCCCTTTGAATCAAGGCGGACTGAGTTATGGATG \| LaeA::FLAG tag \| \| FlagriboR \| TGCCACTCAACGCCATTGACTCAG \| LaeA::FLAG tag \| \| laeAFlag3F \| GATCACTGAGTCAATGGCGTTGAGTGGCATAAGAGCAAAAGGCGACCACATCCAGGAACG \| LaeA::FLAG tag \| \| laeAFlag3R \| TGGTGATGGTGAGAAGGATGGG \| LaeA::FLAG tag \| \| laeAFlagconfF \| TTCCTTCCACTGTTCCACTCGG \| LaeA::FLAG tag \| \| flagconfR \| TTTGTCAGGCCTGACGTGATCC \| LaeA::FLAG tag \| \| veAFlag5F \| ACATGGACCCGTACTCCTATCC \| VeA::FLAG tag \| \| veAFlag5R \| GGCTCCAGCGCCTGCACCAGCTCCGGCACCACGCATGGTGGCAGGCTTTGAGACCATCCG \| VeA::FLAG tag \| \| veAFlag3F \| GATCACTGAGTCAATGGCGTTGAGTGGCATCATAGTTCTTGGCGGGTTCTGGTATAGG \| VeA::FLAG tag \| \| veAFlag3R \| TCGTTTCGAAGTTGCGCAAGGG \| VeA::FLAG tag \| \| veAFlagconfF \| GAATTCTTGGAGTTCCGGCTGG \| VeA::FLAG tag \| |
| --- | --- | --- | --- | --- | --- | --- | --- | --- | --- | --- | --- | --- | --- | --- | --- | --- | --- | --- | --- | --- | --- | --- | --- | --- | --- | --- | --- | --- | --- | --- | --- | --- | --- | --- | --- | --- | --- | --- | --- | --- | --- | --- | --- | --- | --- | --- | --- | --- |

**References**

1. Tsitsigiannis DI, Kowieski TM, Zarnowski R, Keller NP. 2004. Endogenous lipogenic regulators of spore balance in *Aspergillus nidulans*. Eukaryot Cell 3:1398–1411. https://doi.org/10.1128/EC.3.6.1398-1411.2004.
2. Bok JW, Soukup AA, Chadwick E, Chiang YM, Wang CC, Keller NP. 2013. VeA and MvlA repression of the cryptic orsellinic acid gene cluster in *Aspergillus nidulans* involves histone 3 acetylation. Mol Microbiol 89:963–974. https://doi.org/10.1111/mmi.12326.
3. Bayram O, Krappmann S, Ni M, Bok JW, Helmstaedt K, Valerius O, Braus-Stromeyer S, Kwon NJ, Keller NP, Yu JH, Braus GH. 2008. VelB/VeA/LaeA complex coordinates light signal with fungal development and secondary metabolism. Science 320:1504–1506. https://doi.org/10.1126/science.1155888.
